# KRAS(G12C)–AMG 510 interaction dynamics revealed by all-atom molecular dynamics simulations

**DOI:** 10.1038/s41598-020-68950-y

**Published:** 2020-07-20

**Authors:** Tatu Pantsar

**Affiliations:** 1 Department of Pharmaceutical and Medicinal Chemistry, Institute of Pharmaceutical Sciences, Eberhard Karls University Tübingen, Tübingen, Germany; 2 School of Pharmacy, University of Eastern Finland, Kuopio, Finland

**Keywords:** Computational biophysics, Drug development, Targeted therapies, Computational biology and bioinformatics, Structural biology, Molecular modelling, Drug discovery

## Abstract

The first KRAS(G12C) targeting inhibitor in clinical development, AMG 510, has shown promising antitumor activity in clinical trials. On the molecular level, however, the interaction dynamics of this covalently bound drug–protein complex has been undetermined. Here, we disclose the interaction dynamics of the KRAS(G12C)–AMG 510 complex by long timescale all-atom molecular dynamics (MD) simulations (total of 75 μs). Moreover, we investigated the influence of the recently reported post-translational modification (PTM) of KRAS’ N-terminus, removal of initiator methionine (iMet1) with acetylation of Thr2, to this complex. Our results demonstrate that AMG 510 does not entrap KRAS into a single conformation, as one would expect based on the crystal structure, but rather into an ensemble of conformations. AMG 510 binding is extremely stable regardless of highly dynamic interface of KRAS’ switches. Overall, KRAS(G12C)–AMG 510 complex partially mimic the native dynamics of GDP bound KRAS; however, AMG 510 stabilizes the α3-helix region. N-terminally modified KRAS displays similar interaction dynamics with AMG 510 as when Met1 is present, but this PTM appears to stabilize β2–β3-loop. These results provide novel conformational insights on the molecular level to KRAS(G12C)–AMG 510 interactions and dynamics, providing new perspectives to RAS related drug discovery.

## Introduction

KRAS is a driver oncogene that is observed particularly in pancreatic, colorectal and lung cancers^1^. Most often, KRAS becomes oncogenic by a missense mutation in codon 12 which is coding glycine (G12). From the KRAS G12 missense mutants, G12D and G12V are the most frequent, followed by G12C at the third place^2^. In lung cancers, however, smoking-associated G12C mutation is dominating^3,4^. Overall, there is a high demand for mutant KRAS targeted therapies^5,6^.

As a noncatalytic cysteine, G12C is a suitable target for pharmacological intervention via a covalent inhibition of a small molecule drug^7^, providing a potential strategy to target this oncogenic KRAS mutant protein. Since discovery of an allosteric pocket beneath switch-II^8^, determined efforts have been made in the development of G12C targeting covalent inhibitors. Few years later from this initial discovery, a cell-active G12C targeting covalent inhibitor was disclosed^9^, and finally in vivo activity was demonstrated with ARS-1620^10^. For more comprehensive overview of covalently reacting G12C targeting small molecules, the reader is recommended a recent review by Goody et al*.*^11^.

**Figure 1:**
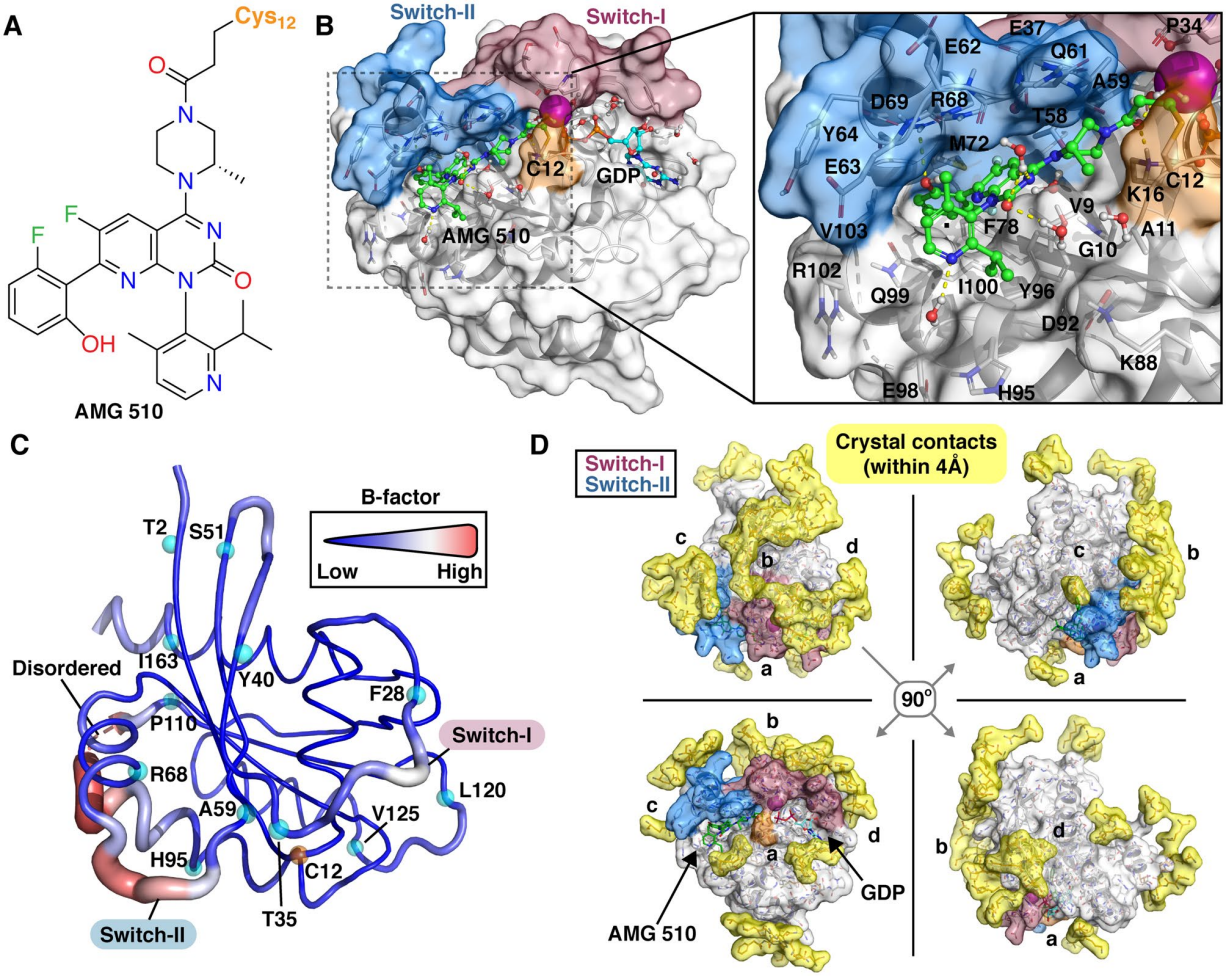
KRAS(G12C)–AMG 510 structure. (**A**) Chemical structure of AMG 510. (**B**) Crystal structure of KRAS(G12C)–AMG 510 complex (PDB ID: 6oim). AMG 510 (green) binds to the SII-P and exploits a cryptic pocket formed by H95/Y96/Q99. (**C**) *B*-factor values of KRAS(G12C)–AMG 510 structure. Thick and red ribbon indicate a high *B*-factor value, whereas thin and blue a low value. Cα-atoms of start and end residues of higher *B*-factor regions are highlighted with cyan spheres (regions: G0–T2; F28–T35; Y40–S51; A59–R68; H95–P110; L120–V125; I163–H166). A disordered region of the structure, D105–E107, is indicated with dashed red line. (**D**) Observed crystal contacts in KRAS(G12C)–AMG 510 structure. Residues within 4 Å of the protein are shown in sticks together with their molecular surface (yellow). In (**B**, **D**) KRAS switch regions: switch-I: residues 25–40 and switch-II: residues 58–72 are highlighted with red and blue, respectively. C12 is highlighted with orange.

Currently, G12C inhibitors have reached the clinical trials. The first covalent drug in the clinical development, AMG 510 (Fig. 1A), demonstrated promising antitumor activity^12^. Another G12C targeting inhibitor, for which clinical data has been reported, is MRTX849 from Mirati Therapeutics^13^. Overall, four KRAS(G12C) targeting inhibitors are currently in the clinical trials. In addition to AMG 510, which is in Phase 1/2 as monotherapy (NCT03600883) and in Phase 1 as combination therapy (NCT04185883^14^) and MRTX849 in Phase 1/2 (NCT03785249), Johnson & Johnson’s G12C covalent inhibitor JNJ-74699157 (also known as ARS-3248) is in Phase 1 as monotherapy (NCT04006301) and Eli Lilly and Company’s LY3499446 is in Phase 1/2 studies as mono- and combination therapy (NCT04165031).

From the covalent inhibitors in the clinical development, AMG 510 in complex with KRAS was the first structure that was made publicly available (Fig. 1B) (PDB ID: 6oim)^12^. The compound is binding to switch-II pocket (S-IIP) and specifically exploits a smaller sub-pocket formed by H95, Y96 and Q99 with isopropyl substituent of the *p*-methylpyridine. The KRAS(G12C)–AMG 510 complex is a high-resolution structure (1.65 Å) where both switch regions are fully ordered. In this structure, as with majority of KRAS(G12C) structures with covalently bound ligand, all native cysteine residues are mutated (C51S; C80L; C118S)^15^. In fact, there is only three of KRAS(G12C) covalent inhibitor complex structures available in the Protein Data Bank without any engineered mutations (PDB IDs: 5v71^16^; 5v9l^17^; 5v9o^17^). To note, the effect of these engineered mutations to KRAS conformational dynamics is unclear to date. The KRAS(G12C)–AMG 510 structure also contains an additional glycine (G0) residue at its N-terminal.

In KRAS(G12C)–AMG 510 structure, atomic displacement parameter, also known as *B*-factor that indicates the atomic fluctuations in the crystal^18^, displays the highest values in switch-II and at the end of the helix-α3, which is connected to a disordered loop region D105–E107 (Fig. 1C). Conversely, based on *B*-factor the end-region of switch-I, interswitch region and beginning of switch-II appear relatively stable. Crystal contacts, however, may have influence on switch conformations and stability, which is in fact a commonly shared characteristics among KRAS structures that display ordered switches^15^. Indeed, a closer inspection revealed crystal contacts on top of the stabilized switch regions (Fig. 1D). This suggest possibility that the crystal contacts may play a decisive role for the observed switch conformations in the KRAS(G12C)–AMG 510 structure.

Ligand binding may change the free energy landscape of its target protein, which defines conformations and their frequency that the protein populates^19^. Discrepancy in KRAS conformational dynamics for G12C targeting covalent inhibitors was revealed by hydrogen/deuterium-exchange mass spectrometry (HDX MS)^16^. In the study, Westover et al*.* demonstrated that chloro-hydroxy aniline and quinazoline inhibitors exhibit unique influence on KRAS conformational dynamics. To date, however, no data has been reported of how AMG 510 influences on KRAS dynamics.

Recently, a post-translational modification (PTM) of KRAS, N acetylation of T2 with the excision of M1 (hereafter shortened as NAc) has gained more attention^20–22^. Based on the published structures by Dharmaiah et al.^20^, acetylation of the T2 is crucial for stabilizing KRAS conformation when the initiator M1 is excised from the structure. The acetyl group displays a water mediated contact to residues located in β2–β3-sheets in the interswitch region. No MD simulations of KRAS with this PTM have been reported; therefore, its influence on KRAS dynamics is still somewhat unclear.

Here, we utilized long timescale MD simulations to investigate the dynamic interactions of the KRAS(G12C)–AMG 510 complex. Our simulations suggest, in contrast with what is observed in crystal structure of the KRAS(G12C)–AMG 510 complex, that the switches are not in a closed defined configuration but rather appear in an ensemble of conformations. Furthermore, we investigated for the first-time the effect of the N-terminal PTM on KRAS dynamics by MD simulations. Interestingly, our data suggests that this PTM further stabilizes KRAS in the interswitch β2–β3-loop compared to KRAS with M1.

## Results

### KRAS(G12C)–AMG 510 interactions in the dynamic environment

**Figure 2:**
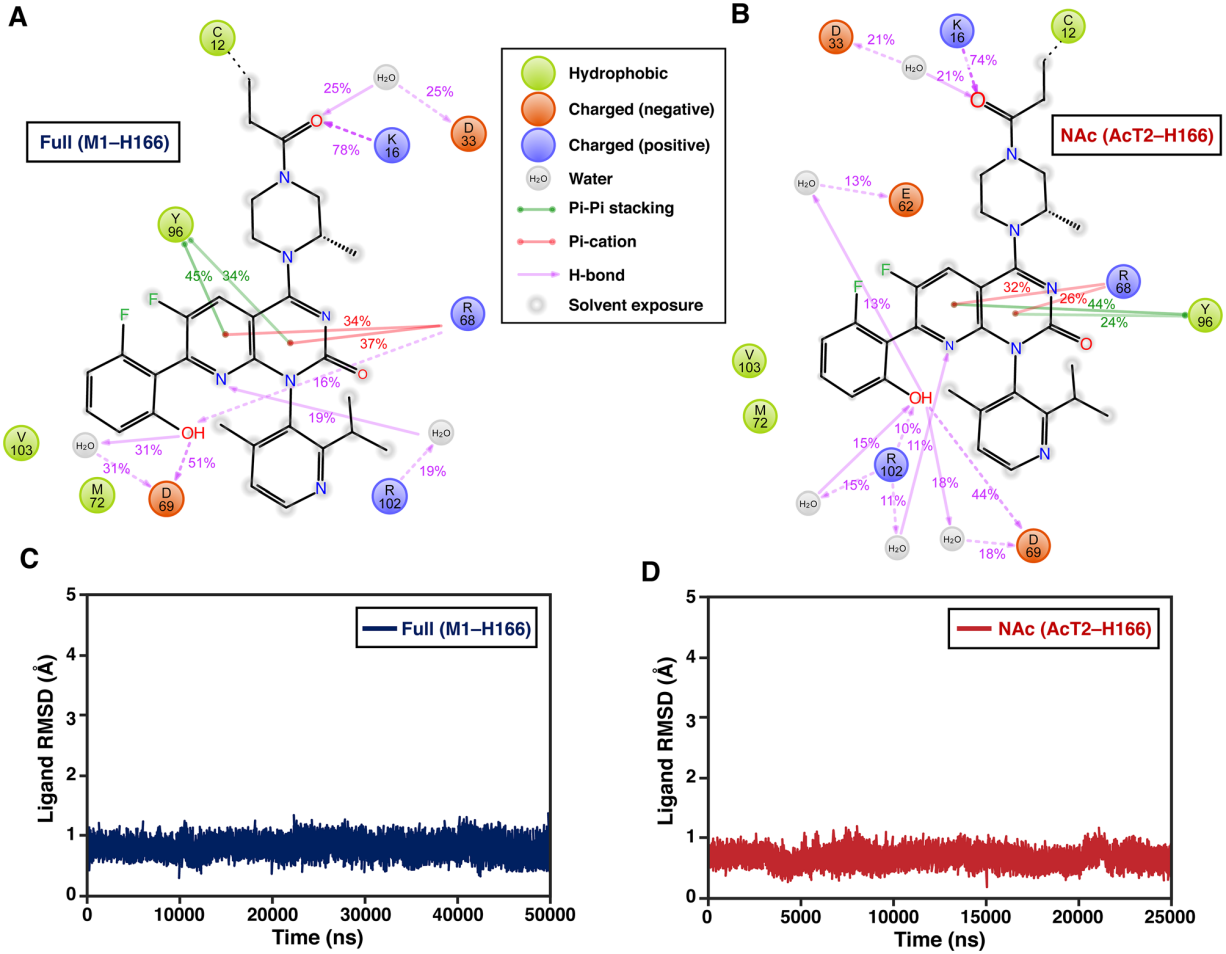
KRAS(G12C)–AMG 510 interactions in the MD simulations. (**A**) Observed KRAS(G12C)–AMG 510 interactions with the simulations of Full (M1–H166) systems (total of 50 μs simulation data). (**B**) Observed KRAS(G12C)–AMG 510 interactions with the simulations of NAc (AcT2–H166) systems (total of 25 μs simulation data). Interactions that occur more than 10% of the simulation time are displayed. (**C**) Ligand root-mean-square deviation (RMSD) in the simulations of Full (M1–H166) systems. (**D**) Ligand RMSD in the simulations of NAc (AcT2–H166) systems.

First, we evaluated how well KRAS(G12C)–AMG 510 interactions observed in the crystal structure reflect to the ones observed in dynamic environment (Supplementary Fig. S1; Fig. 1B). Overall, the interactions observed in the crystal structure are well maintained throughout the MD simulations of *Full* (M1–H166) systems (Fig. 2A). Similar interaction profile is also displayed with N-terminally modified *NAc* (AcT2–H166) systems (Fig. 2B). However, dissimilarities in interactions between the crystal structure and MD simulations exist. Remarkably, methylpiperazine linker appears solvent exposed and is not shielded from water by switch-II (Fig. 2A,B), while in the crystal structure it is enclosed beneath the switch-II (Fig. 1B; Supplementary Fig. S1). In addition to the interaction with K16, the carbonyl-oxygen of AMG 510 next to the covalent attachment point displays a water bridged interaction to residue D33 in switch-I (Fig. 2A,B). This reveals a surprising possibility for AMG 510 to interact with switch-I. This interaction is displayed in two out of five replicate simulations with both systems (Supplementary Figs. S2–S3). Hydroxyl group of AMG 510, which do not present any direct polar interaction in the crystal structure, displays several possibilities for polar interactions, as water bridged interactions to E62, D69 or R102 and direct H-bonds with D69 or R102 are observed.

AMG 510 exploits so-called cryptic pocket in SII-P, which is formed by the residues H95/Y96/Q99^23^. From these residues, Y96 forms π–π interactions with azaquinozoline and these interactions are well conserved throughout the simulations (Fig. 2A,B); whereas Q99 and H95 form more unspecific water bridges (Supplementary Figs. S2–S3). Furthermore, from this heteroaromatic azaquinozoline a cation–π interaction is occurring with R68 of switch-II.

In the energy-minimized crystal structure, carbonyl group of the azaquinozoline displays interactions to two water molecules, one forming a putative water bridge to Q61. However, throughout the simulations no conserved interactions are observed for this carbonyl oxygen. Similarly, from the polar heteroatoms of AMG 510, aromatic nitrogens in the solvent interface are not displaying any conserved interactions during simulations, whereas pyridine nitrogen interacts with water in the crystal structure. Nevertheless, their role to interact with water is quite evident due to their location on solvent interface. These short-lived not-conserved interactions indicate that solvent is disordered in this AMG 510 interface.

Regardless of the increased solvent exposure compared to the crystal structure, AMG 510 is extremely stable throughout the simulations (Fig. 2C,D, Supplementary movies). This implies that a closed switch configuration is not decisive for the compound stability at the SII-P binding site.

### AMG 510 influences on KRAS dynamics

**Figure 3:**
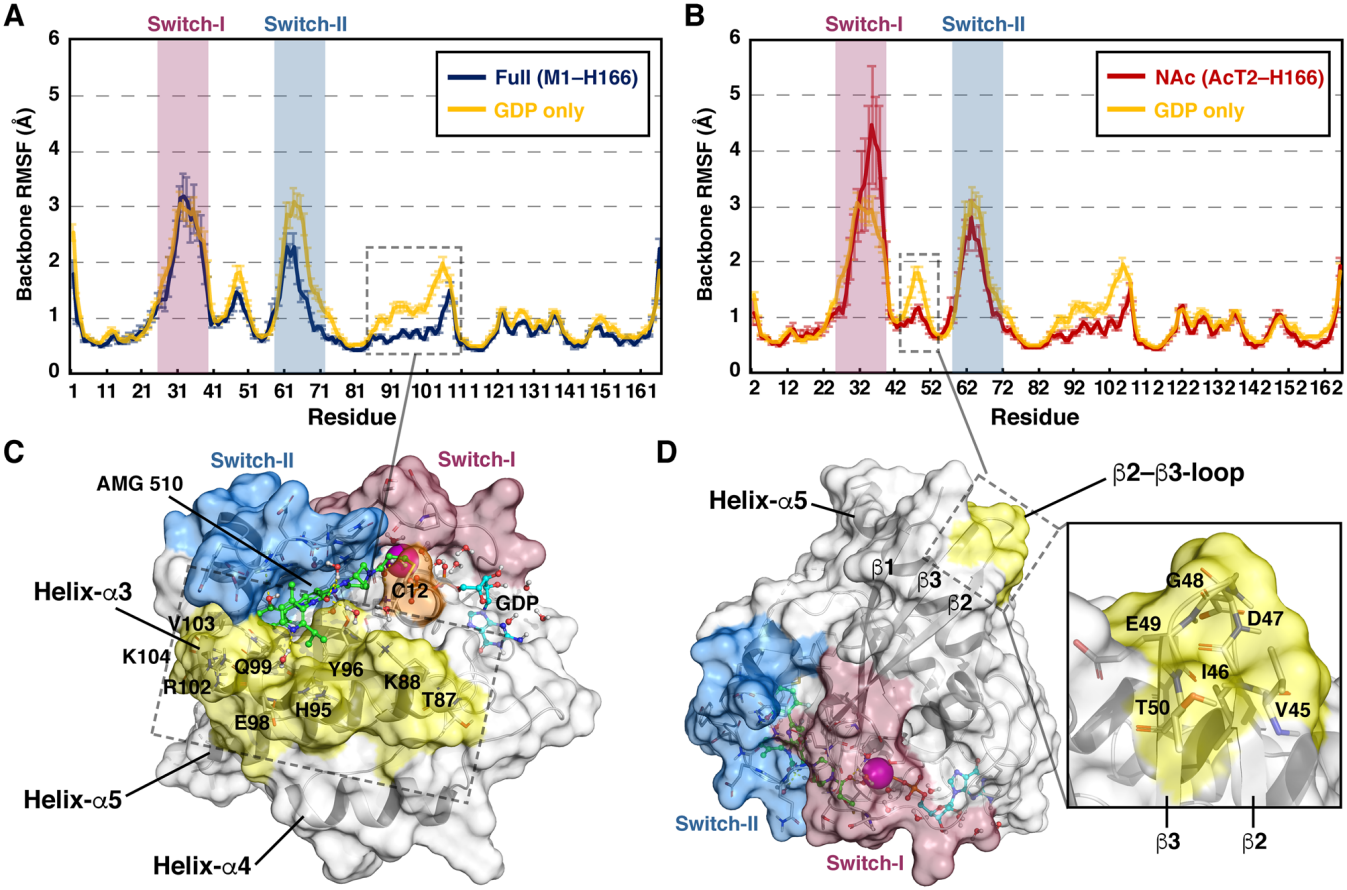
Root-mean-square fluctuations (RMSF) of protein backbone. (**A**) RMSF of full M1–H166 systems (50 μs simulation data). (**B**) RMSF of NAc (AcT2–H166) systems (25 μs simulation data). The ‘GDP only’ RMSF data in **A** and **B** is taken from previously published 70 μs KRAS–GDP simulations^2^. Error bars represent standard error. (**C**) Lower RMSF values are observed in both systems for the Helix-α3, which location and residues are highlighted in yellow (PDB ID: 6oim). (**D**) Lower RMSF values are observed with NAc systems for the β2–β3-loop, which location and residues are highlighted in yellow (PDB ID: 6oim).

Next, we next ascertained if AMG 510 has an influence on KRAS protein dynamics. Based on the protein backbone root-mean-square fluctuation (RMSF) values, overall dynamics of AMG 510 KRAS complex appears similar as observed previously for GDP bound KRAS in long timescale simulations^2^ (Fig. 3). However, differences are also evident. Switch-II region fluctuations are alleviated compared to values observed for KRAS bound to GDP, indicating a stabilization of this switch by AMG 510 (Fig. 3A). Yet, this switch-II stabilization is not so evident with NAc systems (Fig. 3B). Residues forming the cryptic H95/Y96/Q99 pocket are remarkably stabilized in both systems. Moreover, AMG 510 not only stabilizes these pocket residues with direct interactions, but also the whole α3-helix and part of the loop after the helix (residues N86–E107) (Fig. 3C). This stabilization, however, is lower in NAc systems compared to Full systems, but still exists. Also, NAc systems exhibit a trend of higher fluctuation in the end-part of switch-I (D33–S39) compared to GDP only or Full systems. In contrast, NAc systems display remarkable stability in the loop between the β2 and β3 sheets (β2–β3-loop) (Fig. 3D). This observation suggests a clear influence from this PTM to the dynamics of β2–β3-loop in the interswitch region.

### AMG 510 do not stabilize KRAS switches in the crystal conformation

**Figure 4:**
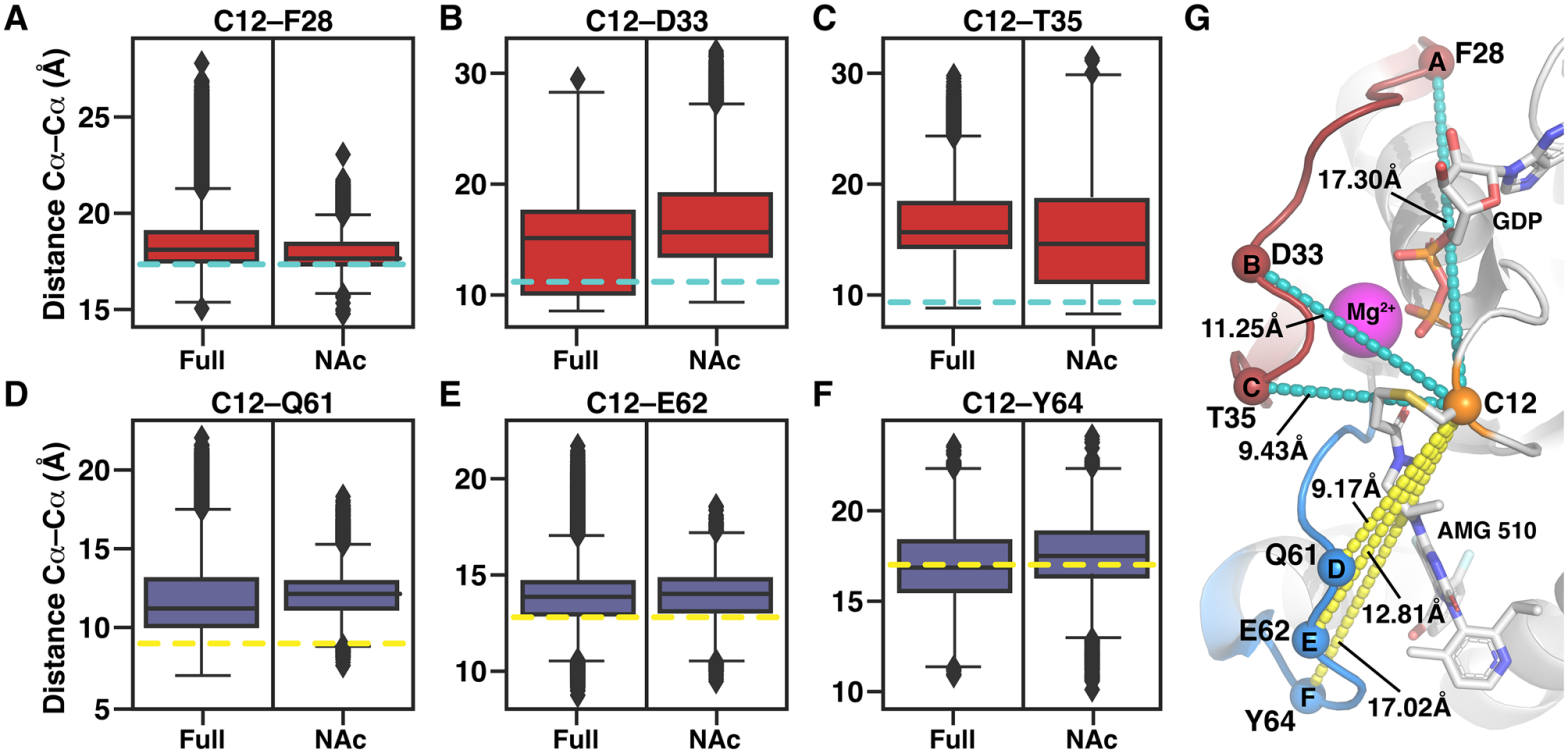
Distances of switch regions to selected switch-I (**A**–**C**) and switch-II (**D**–**F**) residues. The corresponding locations of the monitored Cα atoms (spheres) and their distances in the AMG 510 crystal structure (PDB ID: 6oim) are illustrated with cyan and yellow dashed lines in **A**–**F**. The black horizontal line in the box represents the median. Box displays the quartiles of the dataset (25%–75%) and whiskers the rest of the data with maximum 1.5 IQR. Outliers are indicated with black diamonds. (**G**) Monitored distances represented in the KRAS(G12C)–AMG 510 structure.

Based on the RMSF values, switch regions of KRAS appear highly flexible. Next, we further examined locations of these regions in simulations compared to the crystal structure. To this end, we monitored the distances from C12 to selected reference residues: F28; D33 and T35 in switch-I and Q61; E62 and Y64 in switch-II (Fig. 4). Compared to KRAS(G12C)–AMG 510 crystal structure, simulations indicate clearly higher distances especially in the connection interface of the switches: in end of switch-I and beginning of switch-II. This is demonstrated by higher distances of C12–T35 and C12–Q61 (Fig. 4C,D). In contrast, distances from C12 to the beginning of switch-I (F28) and to the end of switch-II (Y64) are in better agreement with the crystal structure. These results indicate that the switches clearly prefer more open conformations, particularly in the connection interface of the switches.

### KRAS(G12C)–AMG 510 complex populate an ensemble of conformations

Switch regions display highly dynamical characteristics in the MD simulations that deviate from the observed KRAS(G12C)–AMG 510 crystal structure conformation. Based on the data presented above, switches prefer more open states compared to the crystal structure; however, with ambiguous configurations. To obtain more comprehensive information of their conformational distribution, we utilized Markov state modelling (MSM) approach^24,25^. MSM is an optimal method to disclose relevant conformational states of a biomolecule from a long timescale simulation data.

**Figure 5:**
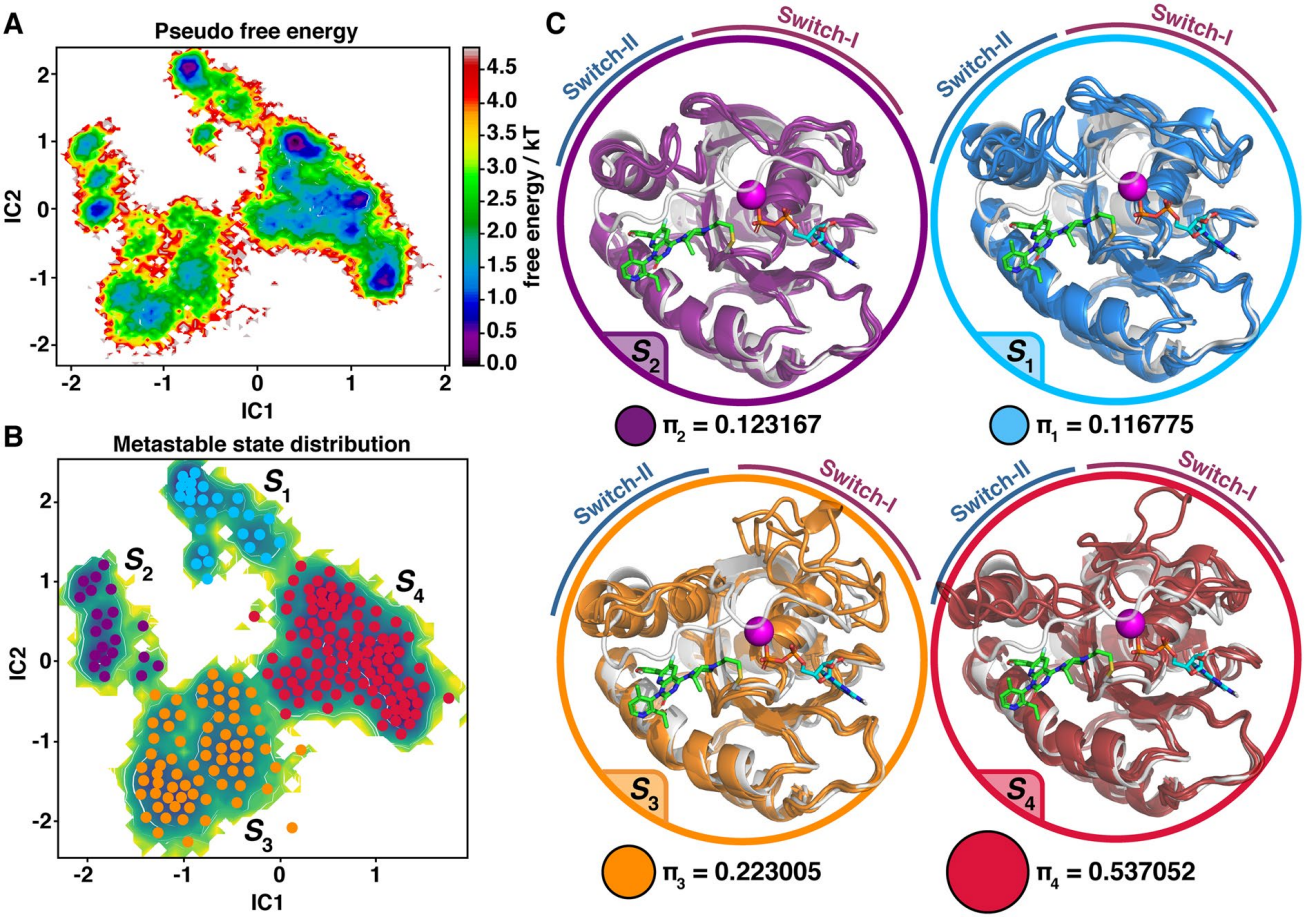
Metastable states of KRAS(G12C)–AMG 510 complex revealed by Markov state modelling. (**A**) Pseudo free energy map of distributions along time-lagged independent components (ICs) 1 and 2. (**B**) Separation of four metastable states (***S***_**1**_–***S***_**4**_) by PCCA++ analysis. (**C**) Visualization of the metastable states. Each metastable state (***S***_**i**_) is illustrated with three representative structures (coloured cartoons) and the crystal structure conformation of KRAS(G12C)–AMG 510 complex is shown as a reference in grey. Equilibrium probability (**π**_**i**_) for each state is indicated below the conformations together with circles with an area that is relative to state probability.

MSM of Full (M1–H166) systems revealed four metastable states (***S***_**1**_–***S***_**4**_) for the KRAS(G12C)–AMG 510 complex (Fig. 5). Overall, it is quite evident that all these states deviate from the crystal structure conformation (Fig. 5C). In metastable states ***S***_**1**_ and ***S***_**2**_, switch-II is forming a helical conformation that is in orientation perpendicular to α2-helix. This type of helical switch-II configuration is observed in many published KRAS crystal structures (Supplementary Fig. S4) (*e.g.* PDB IDs: 6quu^26^; 6gj7^27^; 6mqg^28^; 6bof^29^; 6qux^26^; 6quw^26^; 6quv^26^). Overall these ***S***_**1**_ and ***S***_**2**_ conformations appear similar; however, with ***S***_**2**_ switch-I is in more open conformation at the beginning of the switch (residues D30–D33) and also α2-helix is more stable with this state.

In the second most populated state ***S***_**3**_, the end-part of switch-I (starting from T35) exists in a fully open conformation (Fig. 5C). This conformation resembles the KRAS conformation observed in SOS1–KRAS complex (Supplementary Fig. S5) (PDB ID: 6epl)^30^. In fact, this might be a native pre-bound configuration of switch-I for GDP-bound KRAS that is required for SOS1 binding and facilitates the nucleotide exchange. Furthermore, ***S***_**3**_ displays a specific helical configuration at the beginning of switch-II (D57–Q61).

The most populated metastable state ***S***_**4**_ exhibits a loop-like configuration of switch-II. The switch-I conformation displays higher variability within this metastable state compared to other states, as both closed and open conformations are observed. These variable switch-I configurations are probably reflecting to the observed multiple energy minima that are included within this state (Fig. 5A).

Transitions between these metastable states occur in microsecond timescale, where mean-first-passage-time of transitions vary between 1.76 μs (from ***S***_**3**_ to ***S***_**4**_) and 16.79 μs (from ***S***_**4**_ to ***S***_**2**_) (Supplementary Fig. S6).

## Discussion

Our simulations reveal novel atomic scale insights of a KRAS(G12C) targeting inhibitor in the clinical development. Surprisingly, AMG 510 does not fix KRAS switches to a closed conformation as would be expected based on the crystal structure. Instead, in the simulations this complex exists in an ensemble of open conformations, confirmed by MSM. In fact, KRAS(G12C)–AMG 510 complex appears to follow generally the native dynamics of GDP-bound KRAS. The observed conformations for the switches in the KRAS(G12C)–AMG 510 crystal structure are most likely induced by neighbouring proteins in the crystal environment (crystal contacts). This is indeed a common feature observed in all KRAS structures that display ordered switch conformations^15^. These open states of KRAS switches have been also observed in other long timescale simulations with a membrane, where a different force field was used^31^.

Regardless of the switch fluctuations, AMG 510 appears extremely stable throughout the simulations. This occurs even though the switches are not fully shielding AMG 510 from solvent. This implies that an inhibitor bound to SII-P needs to be optimized for a dynamic environment. In fact, the observed switch fluctuations that occur even when an inhibitor is bound, may provide a putative explanation for difficulties in interpretation of structure–activity-relationship (SAR) with KRAS binding ligands. Additional interesting feature is that through a water bridged interaction to D33, AMG 510 can have direct connection and influence on switch-I. This adds an additional layer of complexity in KRAS–inhibitor interactions.

Although switch regions of KRAS are highly dynamic, other parts of the protein appear more fixed. Especially the cryptic pocket that is exploited by AMG 510 is extremely stable throughout the simulations. Interestingly, stabilization of this pocket by AMG 510 further stabilizes KRAS dynamics in the whole α3-helix region. To note, this region may play an important role in KRAS dimerization and nanoclustering^32–34^.

One metastable state of KRAS(G12C)–AMG 510, state ***S***_**3**_, resembles a pre-nucleotide exchange conformational state. Although switch-II conformation in this state when an inhibitor is bound to SII-P disallows binding of SOS1, it may be important for the efficacy of an inhibitor to allow KRAS to visit these native conformations.

To the best of our knowledge, we report here for the first-time MD simulations with N-terminally processed KRAS. As shown by Dharmaiah et al.^20^, N-acetylation of T2 stabilizes KRAS dynamics upon the excision of M1. Here with AMG 510, dynamics of full N-term with iMet and N-acetylated (T2) appear also comparable, agreeing with the reported structural observations. Furthermore, the interaction profile of AMG 510 is similar with or without this PTM (Fig. 2). Therefore, N-terminal modification of KRAS is not expected to affect binding of S-IIP inhibitors, as is clearly demonstrated with the observed efficacy of AMG 510^12,23^. Nevertheless, this PTM appears to stabilize KRAS dynamics in the loop region that connects beta-sheets β2 and β3 (residues 45–50). This β2–β3-loop may play a crucial role at KRAS dynamics at the membrane, as based on the ‘exposed’ configuration NMR data driven structure this loop exist in in a direct contact to the membrane (PDB ID: 2msc)^35^. Moreover, in a long timescale simulations study of KRAS at the membrane this orientation was frequently populated^31^. Recent NMR data driven models of KRAS–RAF on nanodiscs suggest that this loop is in contact with the CRD-domain of RAF (PDB IDs: 6pts, 6ptw)^36^. In a study with HRAS, mutations to residues in this region D47A, E49A were shown to enhance RAS nanoclustering and its signalling activity^37^. Also, T50I mutation with NRAS is observed in Noonan syndrome that leads to increased signalling^38^. These results highlight the fact that it is important to take into account the possibility that N-terminal processed KRAS may behave differently at the membrane compared to full-length KRAS with M1 present, which has been used in most of the studies. Therefore, future research should clarify if this N-terminal PTM affects KRAS dynamics at the membrane.

The dynamics here generally follow what has been previously observed for KRAS. As always, in MD simulations the selected methodology could however cause bias to the observed dynamics (see details in methods). For instance, the applied force field here is not a polarizable force field^39^, which could have influence on the results.

Overall, these results provide novel atomic-level insights to KRAS(G12C)–inhibitor complex. First, they suggest that KRAS exists in an ensemble of conformations when AMG 510 is bound. Second, they indicate that a KRAS targeting inhibitor should be optimized to a more solvent exposed dynamic environment then what a crystal structure suggests, as the switches appear dynamic even when an inhibitor is present in SII-P. Finally, potential influence of N-terminal PTM of KRAS at the membrane needs to be clarified in future. Proper understanding of KRAS and its conformational dynamics is crucial, especially when targeting other mutant forms of this oncoprotein which are lacking the cysteine for covalent inhibition.

## Methods

### Molecular dynamics simulations

All the molecular modelling was conducted with Maestro^40^ using OPLS3e force field^41,42^. OPLS3e generated parameters were applied for the ligands, GDP and AMG 510. For the simulations, we utilized the AMG 510′s lead molecule – KRAS complex (PDB ID: 6p8y^43^). Engineered mutations of the structure were reverse mutated back to native wild-type form (C51S; C80L; C118S). The ligand was manually changed to AMG 510 and the complex was optimized and energy-minimized by Protein preparation wizard^44^. Additionally, for the PTM NAc systems, M1 was deleted and the resulting terminal T2 was acetylated.

Simulations were run using Desmond MD engine^45^. These minimized KRAS(G12C)–AMG 510 complexes were solvated to cubic boxes with a minimum distance of 15 Å from the protein. Water was described with TIP3P water model^46^. K^+^-ions and Cl^−^-ions were added to obtain 0.15 M ionic strength with a total net charge neutral. MD simulations were run in NpT ensemble (T = 310 K, Nosé-Hoover method; p = 1.01325 bar, Martyna-Tobias-Klein method) with default Desmond settings. RESPA integrator with 2 fs, 2 fs and 6 fs timesteps were used for bonded, near and far, respectively. The default value of 9 Å was used for Coulombic cutoff.

Five replicate simulation for each system using different seed numbers were run. Full (M1–H166) KRAS(G12C)–AMG 510 simulation were simulated for 10 μs and NAc systems for 5 μs, resulting in total of 50 μs and 25 μs aggregate simulations, respectively. A default Desmond relaxation protocol was applied before the production simulations.

Maestro tools were used for all of the analysis, except for MSM (see MSM details below). Visualization of the structures was done with PyMOL^47^.

### Markov state model generation

MSM generation was conducted with PyEMMA 2^48^. Bayesian MSM was conducted following the general recommendations^49^. The individual trajectories of full M1–H166 systems were used as an input for MSM generation. For featurization, we used the backbone torsions of residues from switch-I and switch-II together with selected residues in contact with AMG 510 (residues 25, 26, 27, 28, 29, 30, 31, 32, 33, 34, 35, 36, 37, 38, 39, 40, 58, 59, 60, 61, 62, 63, 64, 65, 66, 67, 68, 69, 70, 71, 72, 95, 96, 99, 103). During the decision process of which residues were selected to be included in the final model, we monitored closely the VAMP-2 score^50^, which was 2.55 for the final model. Dimensional reduction was conducted with time-lagged independent component analysis (TICA)^51^. Lag time τ = 10 ns and two dimensions were selected, where the implied timescales were converged, and further validation was conducted with the Chapman-Kolmogorov validation test (Supplementary Fig S7). Discretization of the data to microstates was done by k-means clustering (√N used for the number of clusters). Finally, a spectral clustering using the Perron-cluster cluster analysis (PCCA++)^52^ was used to assign the microstates to metastable macrostates. Transition-path theory (TPT) was applied to investigate state transitions and the flux between metastable states^53,54^.

## Supplementary information

Supplementary information.

## Data Availability

The datasets generated during and analysed during the current study are freely available in the Zenodo repository, https://doi.org/10.5281/zenodo.3711537 The dataset include original raw-trajectories, movies of the MD simulations and PDB-coordinates for the metastable states.
