# Supplementary material for: KRAS(G12C)–AMG 510 interaction dynamics revealed by all-atom molecular dynamics simulations
Source: Sci Rep. 2020 Jul 20;10:11992. doi: 10.1038/s41598-020-68950-y (PMC7371895; doi:10.1038/s41598-020-68950-y)
Supplement: Supplementary file 1 — Supplementary information. [file 41598_2020_68950_MOESM1_ESM.pdf]

SUPPLEMENTARY INFORMATION

KRAS(G12C)–AMG 510 Interaction Dynamics Revealed by All-atom Molecular Dynamics Simulations.

Tatu Pantsar[1,2,*]

[1] Department of Pharmaceutical and Medicinal Chemistry, Institute of Pharmaceutical Sciences, Eberhard Karls University Tübingen, Tübingen, Germany
[2] School of Pharmacy, University of Eastern Finland, Kuopio, Finland
* tatu.pantsar@uef.fi

Content

Supplementary Figure S1..........2
2D-depiction of observed interactions in KRAS(G12C)–AMG 510 crystal structure.

Supplementary Figure S2..........2
Interaction frequencies observed in individual simulation replicas 1–5 (A–E) of Full (M1–H166) systems.

Supplementary Figure S3..........3
Interaction frequencies observed in individual simulation replicas 1–5 (A–E) of NAc (AcT2–H166) systems.

Supplementary Figure S4..........3
Helical switch-II conformation that is in perpendicular orientation to α2-helix is observed in many KRAS structures.

Supplementary Figure S5..........4
MSM metastable state S_3 exhibits similar switch-I conformation as observed in SOS1–KRAS complex.

Supplementary Figure S6..........5
Transition probabilities among the metastable states.

Supplementary Figure S7..........6
Validation of Markov State Model.

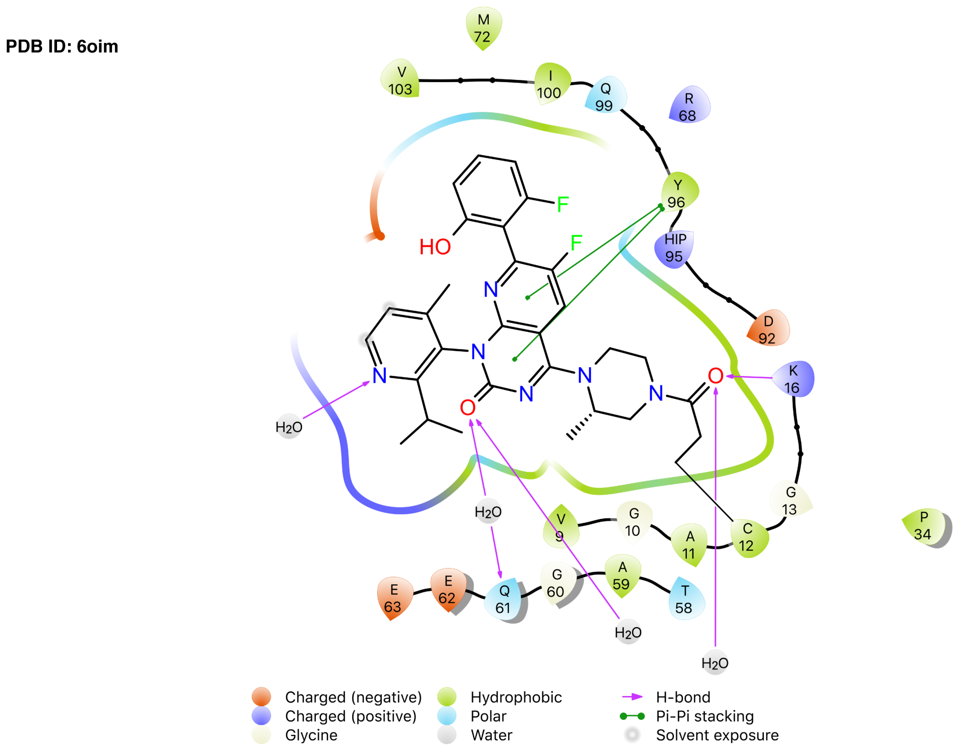

Supplementary Figure S1. 2D-depiction of observed interactions in KRAS(G12C)–AMG 510 crystal structure (PDB ID: 6oim).

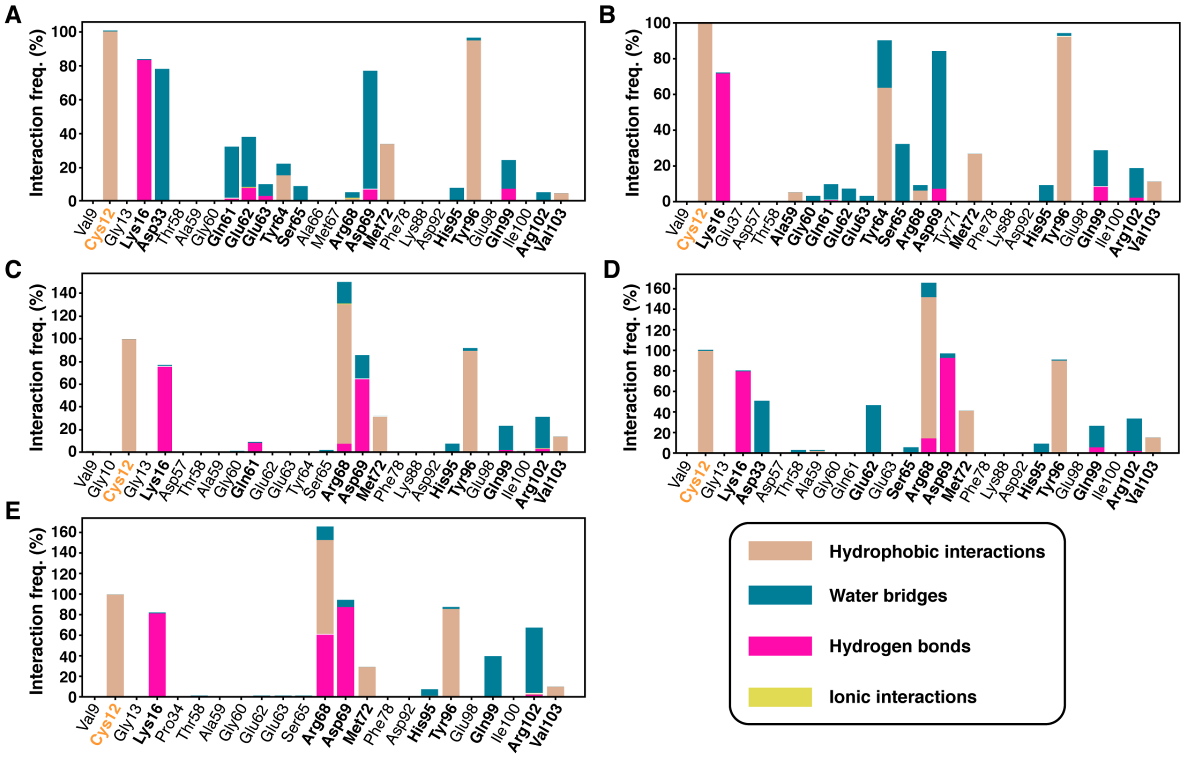

Supplementary Figure S2. Interaction frequencies observed in individual simulation replicas 1–5 (A–E) of Full (M1–H166) systems. Each replica consists of 10 μs MD simulation data.

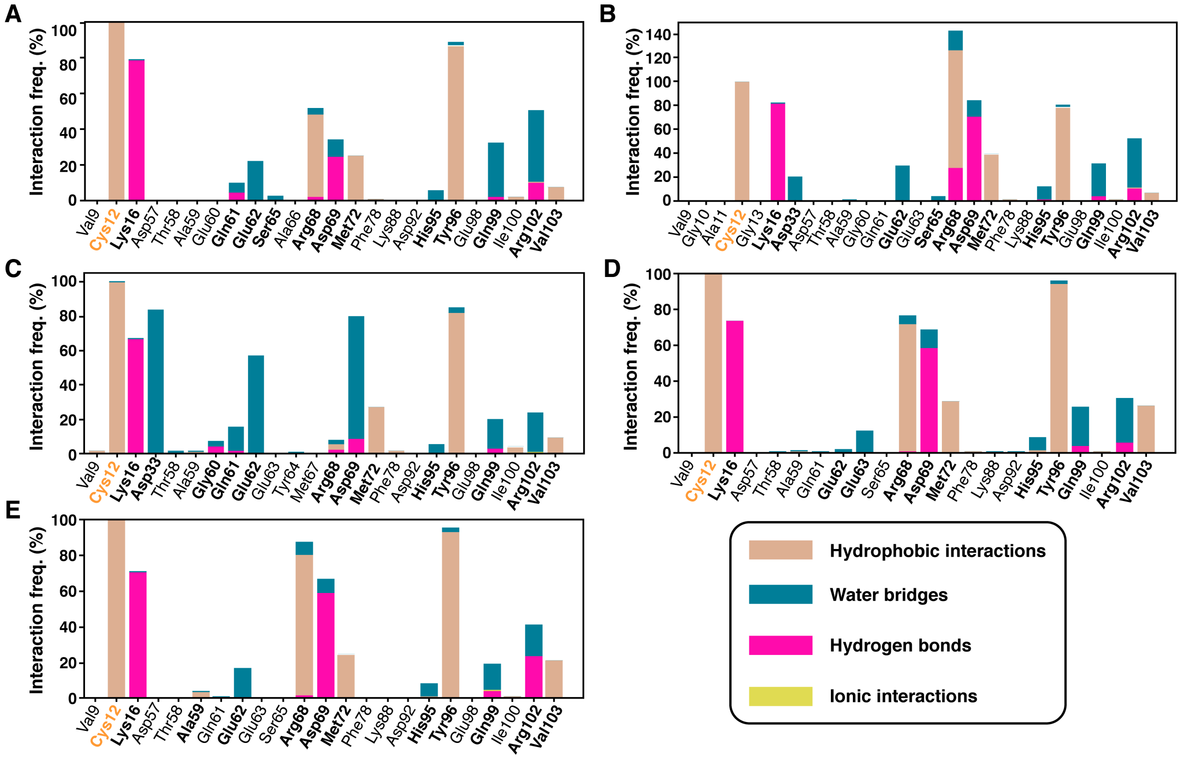

Supplementary Figure S3. Interaction frequencies observed in individual simulation replicas 1–5 (A–E) of NAc (AcT2–H166) systems. Each replica consists of 5 μs MD simulation data.

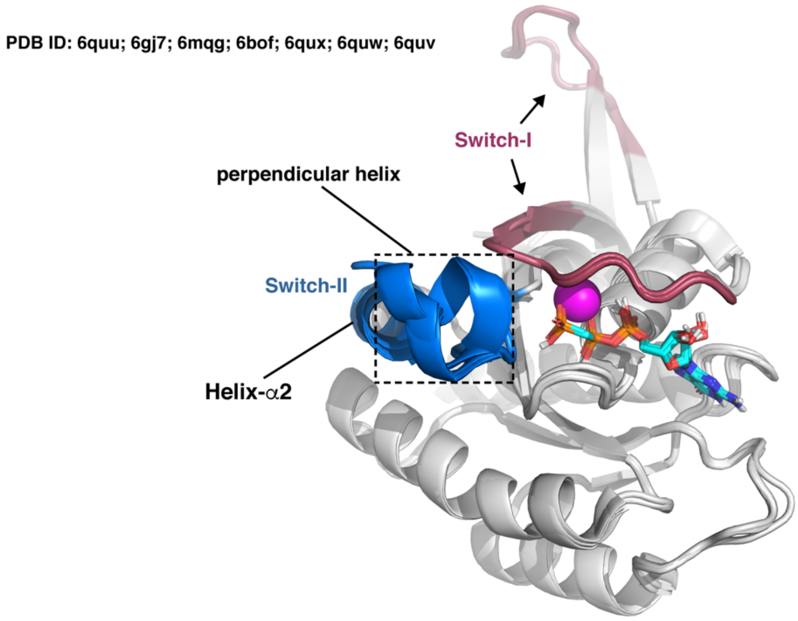

Supplementary Figure S4. Helical switch-II conformation that is in perpendicular orientation to α2-helix is observed in many KRAS structures (PDB IDs: 6quu; 6gj7; 6mqg; 6bof; 6qux; 6quw; 6quv).

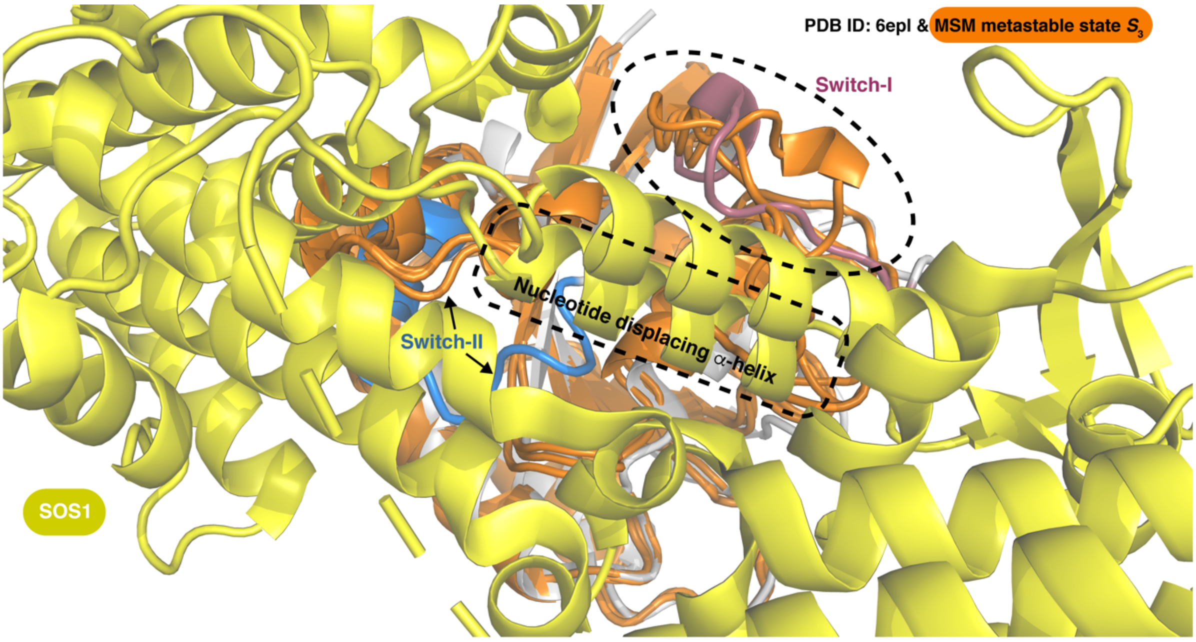

Supplementary Figure S5. MSM metastable state S_3 exhibits similar switch-I conformation as observed in SOS1–KRAS complex. KRAS from the SOS1-KRAS complex (PDB ID: 6epl) is illustrated in grey cartoon with switch-I and switch-II in red and blue, respectively. SOS1 in shown in yellow cartoon. Three conformations of $\mathbf{S_3}$ are illustrated with orange cartoon.

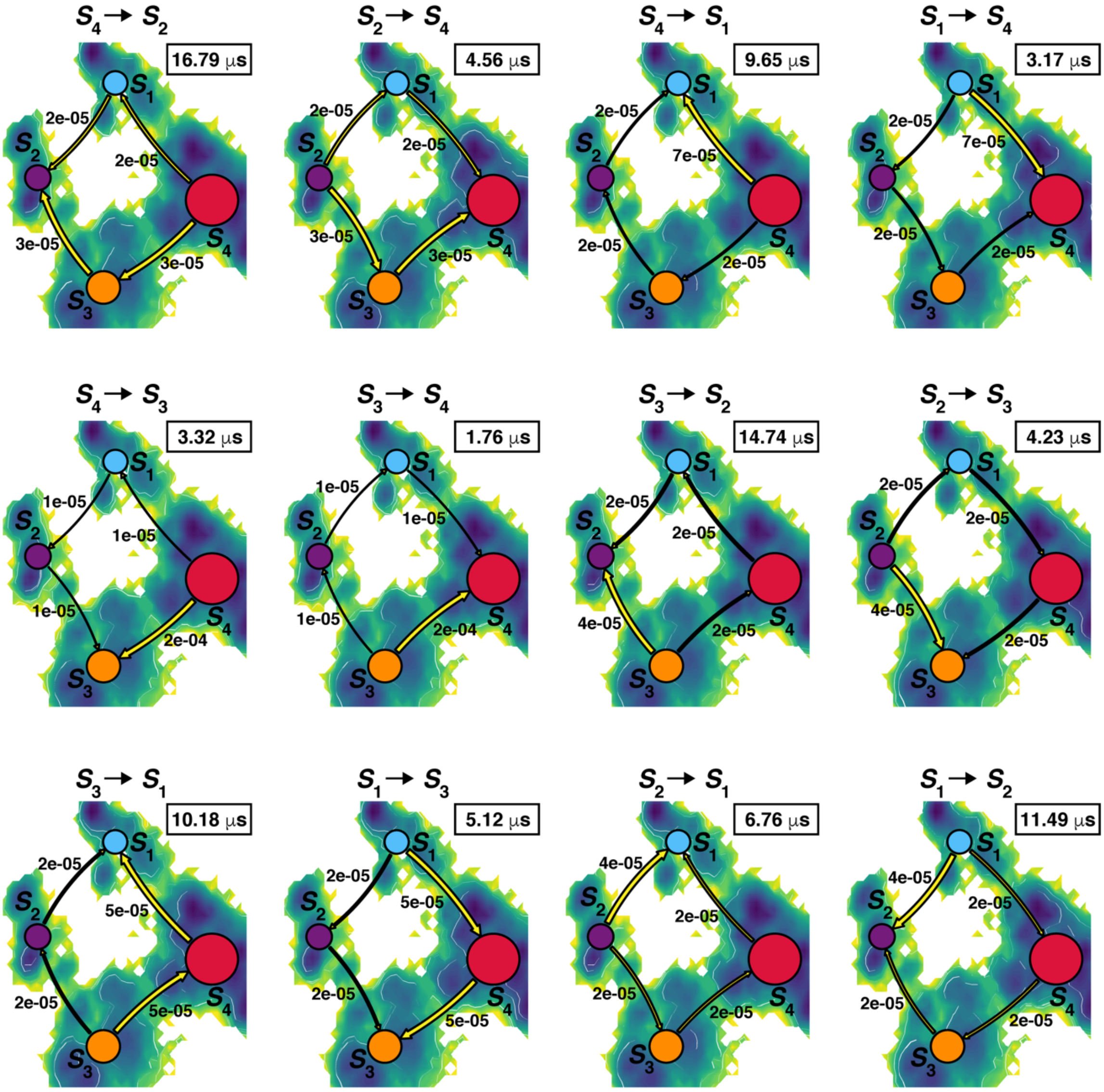

Supplementary Figure S6. Transition probabilities among the metastable states. Conformational change pathways probability flux for each state transition based on transition pathway theory. Mean-first-passage-time of transition is shown for each flux and varies between 1.76 μs and 16.79 μs.

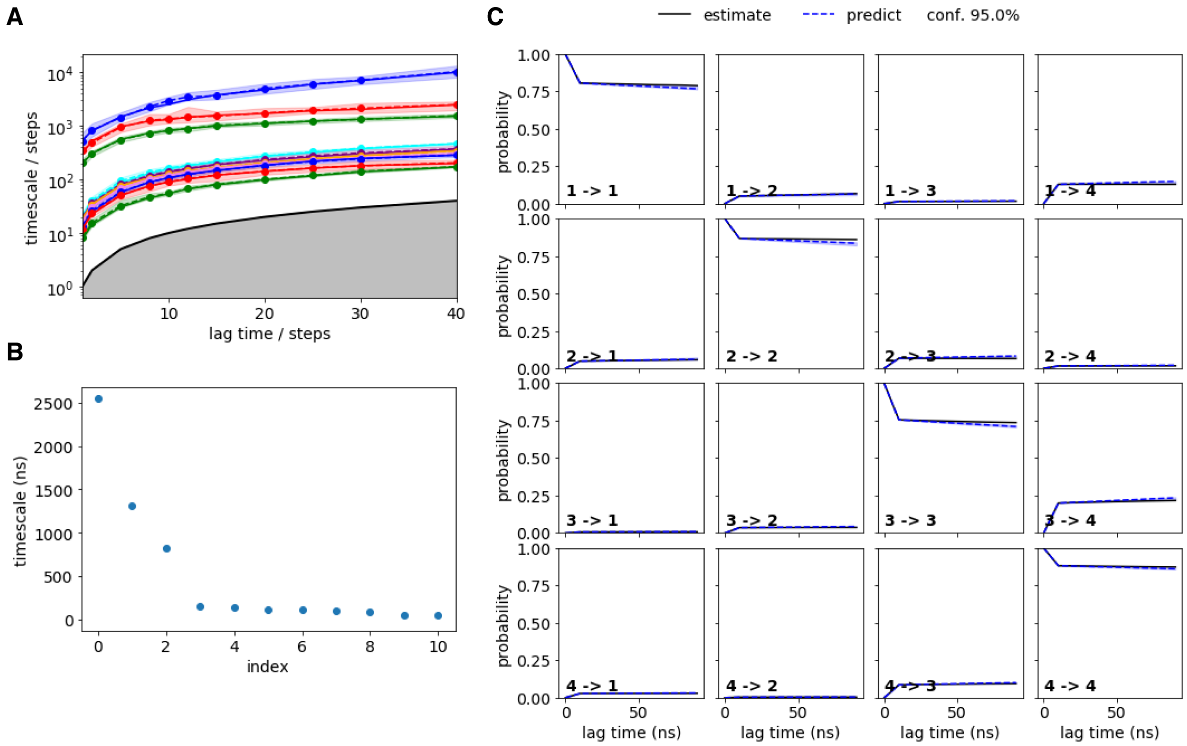

Supplementary Figure S7. Validation of Markov state model. (**A**) Implied relaxation timescales indicate that selected lag time (τ = 10 ns) is suitable. (**B**) Spectrum of relaxation timescales at τ = 10 ns. A clear separation is observed between the third and fourth slowest relaxation times, suggesting a four-state model. (**C**) Chapman-Kolmogorov test of the model indicate accurate prediction of the behaviour on longer timescales.
